# Genetic variation and recombination of RdRp and HSP 70h genes of *Citrus tristeza virus *isolates from orange trees showing symptoms of citrus sudden death disease

**DOI:** 10.1186/1743-422X-5-9

**Published:** 2008-01-16

**Authors:** Clarissa PC Gomes, Tatsuya Nagata, Waldir C de Jesus, Carlos R Borges Neto, Georgios J Pappas, Darren P Martin

**Affiliations:** 1 Programa de Pós-Graduação em Ciências Genômicas e Biotecnologia, Universidade Católica de Brasília. SGAN, Quadra 916, Módulo B, Av. W5 Norte, 70.790-160, Brasília-DF, Brazil; 2 Fundecitrus, Av. Adhemar Pereira de Barros, 201, 14807-040, São Paulo, SP, Brazil; 3 CENARGEN, Parque Estação Biológica, Av. W5 Norte, 70770-900, Brasília, DF, Brazil; 4 Institute of Infectious Disease and Molecular Medicine, University of Cape Town, Observatory, Cape Town, 7000, South Africa; 5 Universidade Federal do Espírito Santo, Centro de Ciências Agrárias, Alto Universitário, S/N, 29500-000, ES, Brazil

## Abstract

**Background:**

Citrus sudden death (CSD), a disease that rapidly kills orange trees, is an emerging threat to the Brazilian citrus industry. Although the causal agent of CSD has not been definitively determined, based on the disease's distribution and symptomatology it is suspected that the agent may be a new strain of *Citrus tristeza virus *(CTV). CTV genetic variation was therefore assessed in two Brazilian orange trees displaying CSD symptoms and a third with more conventional CTV symptoms.

**Results:**

A total of 286 RNA-dependent-RNA polymerase (RdRp) and 284 heat shock protein 70 homolog (HSP70h) gene fragments were determined for CTV variants infecting the three trees. It was discovered that, despite differences in symptomatology, the trees were all apparently coinfected with similar populations of divergent CTV variants. While mixed CTV infections are common, the genetic distance between the most divergent population members observed (24.1% for RdRp and 11.0% for HSP70h) was far greater than that in previously described mixed infections. Recombinants of five distinct RdRp lineages and three distinct HSP70h lineages were easily detectable but respectively accounted for only 5.9 and 11.9% of the RdRp and HSP70h gene fragments analysed and there was no evidence of an association between particular recombinant mosaics and CSD. Also, comparisons of CTV population structures indicated that the two most similar CTV populations were those of one of the trees with CSD and the tree without CSD.

**Conclusion:**

We suggest that if CTV is the causal agent of CSD, it is most likely a subtle feature of population structures within mixed infections and not merely the presence (or absence) of a single CTV variant within these populations that triggers the disease.

## Background

Diseases caused by the aphid-borne *Closterovirus*, *Citrus tristeza virus *(CTV), are among the greatest threats to citrus production worldwide. CTV has a c.a. 19.3 Kb single stranded positive sense RNA genome encoding 19 proteins expressed from 12 open reading frames (ORFs) that is the largest amongst the known closteroviruses [1]. A large amount of phenotypic diversity has been detected amongst CTV isolates characterized from around the world, with the identified strains differing from one another in the symptoms they induce in different citrus genotypes or their aphid transmissibility [2-9].

Since 2001, an emergent disease, called citrus sudden death (CSD), has caused the death or eradication of four million orange trees throughout the Brazilian states of Minas Gerais and São Paulo [10]. The disease is continuing to spread and epidemiological studies indicate that the casual agent is possibly distributed by an air-borne vector [11]. One curiosity of the disease is that it has almost exclusively been reported in trees comprising a sweet orange graft onto a Rangpur lime (*Citrus limonia *Osb.) rootstock. Neither Rangpur lime nor sweet orange appear sensitive to the disease in isolation and diseased trees can sometimes be salvaged by removing the sweet orange graft.

There is some circumstantial evidence implicating CTV as a casual agent of CSD: (1) Trees presenting with CSD symptoms are invariably infected with CTV (Fundecitrus, personal communication); (2) CSD and CTV have similar epidemiological properties [11] and (3) CTV induces symptoms that present and develop in a similar manner to those occurring in trees with CSD [12]. It remains to be confirmed, however, whether CSD is caused by some novel CTV variant, another citrus-infecting virus (for example a marafivirus) [13] or some synergistic interaction between CTV and one or more other infectious agents. Due to its unknown etiology, no serological or molecular tools are available for diagnosing CSD and currently the only reasonably reliable indicator of the disease is yellowing of the phloem vessel system in the tissues of rootstocks.

In an attempt to identify genetic features of CTV that may be associated with CSD, two loci within the RNA-dependent RNA polymerase (RdRp) and the HSP70 homologue (HSP70h) genes were extensively sampled from CTV populations infecting three trees – two with CSD and one without the disease. While significant differences in the population structures of the viruses in the three trees were observed, it was not possible to definitively associate any of these with CSD. While our results demonstrate extraordinary Brazilian CTV genetic diversity and intraspecific recombination within individual plants they also indicate that it is unlikely to simply be the occurrence of a single CTV genetic variant within these mixed CTV infections that causes the disease.

## Results and Discussion

### Within tree CTV populations are unexpectedly diverse

Preliminary analyses of the pairwise genetic distances between the 286 RdRp and 284 HSP70h sequences from the three trees indicated that all were infected with a diverse range of CTV genotypes. Previous analyses of CTV mixed infections have revealed within host CTV population structures that are expected for RNA virus quasispecies: One or two predominant genotypes (>30% of the population) and a number of closely related variants of these [2,6,9]. The CTV populations in the three Brazilian trees analyzed here are extraordinarily diverse by comparison with these previously described populations. For example, the most diverse population of CTV sequences in an individual tree described by Sentandreu et al. [9] was for isolate T385 at the p20 locus. Out of 30 sequences sampled from T385, ten unique haplotypes were identified. The total number of haplotypes identified amongst 451 p20 and 274 p18 sequences sampled from six trees (originating in Spain and Japan) were 35 and 23 respectively. By contrast sequence analysis of the 286 RdRp and 284 HSP70h gene fragments sampled from amongst the three Brazilian orange trees here revealed a total of 76 RdRp and 153 HSP70h haplotypes.

The minimum pairwise sequence identities observed in individual Brazilian trees were 75.9% for two RdRp sequences from tree C3 and 89.0% for two HSP70h sequences from tree C5. In all the trees we observed that RdRp diversity was greater than that of HSP70h. Although the HSP70h gene, which has an important function in virion assembly [14,15], is characteristically more conserved than the RdRp gene, we have paradoxically sampled approximately twice as many HSP70h haplotypes.

### Potential associations between specific CTV variants CSD

We therefore used a mixture of recombination and phylogenetic analyses to characterize the complex populations of CTV RdRp and HSP70h sequences found in the three trees. Since recombination has been previously reported in CTV [2,16] and is a process that violates the assumptions of conventional phylogenetic reconstruction methods (such as neighbor joining, and maximum likelihood) [17], we sought to remove all obviously recombinant sequences from our dataset prior to phylogenetic analysis. We used a battery of recombination analysis methods implemented in RDP3 to identify and remove 17 RdRp and 32 HSP70h sequences that were obviously recombinant.

**Figure 1:**
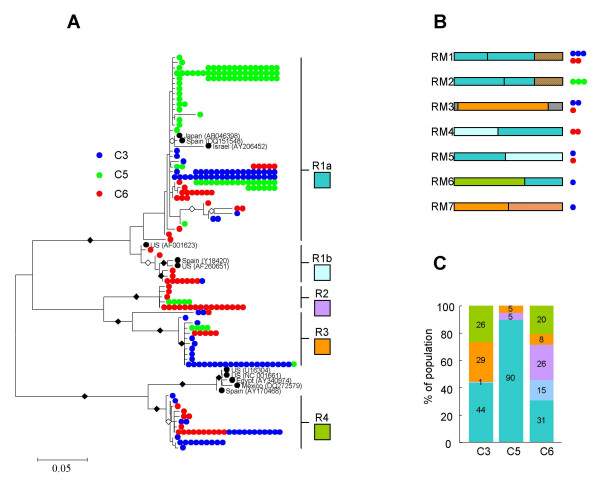
**Phylogenetic and recombination analysis of 286 RdRp gene fragments sampled from three Brazilian orange trees**. (A) Largely recombination free maximum likelihood phylogeny of 269 RdRp gene fragment sequences. Sequences sampled from trees C3, C5 (both with CSD) and C6 (without CSD) are represented by blue, green and red dots respectively. Multiple dots on particular branches represent identical sequences. Whereas branches with more than 75% bootstrap support are labeled with filled black diamonds, those with between 50 and 74% support are labeled with unfilled black diamonds. Sequence lineages R1a, R1b, R2, R3 and R4 are indicated. (B) The mosaic structures of obviously recombinant sequences excluded from the phylogenetic analysis. The mosaics are colour coded according to the main sequence lineages from which they have probably been derived. Grey areas represent sequence tracts that were not clearly derived from one of the five identified sequence lineages in A. Coloured dots beside the schematic representations of the recombinant sequences represent the numbers and origins of recombinant sequences sharing similar mosaics. (C) The relative population representation of different major RdRp lineages identified in A.

**Figure 2:**
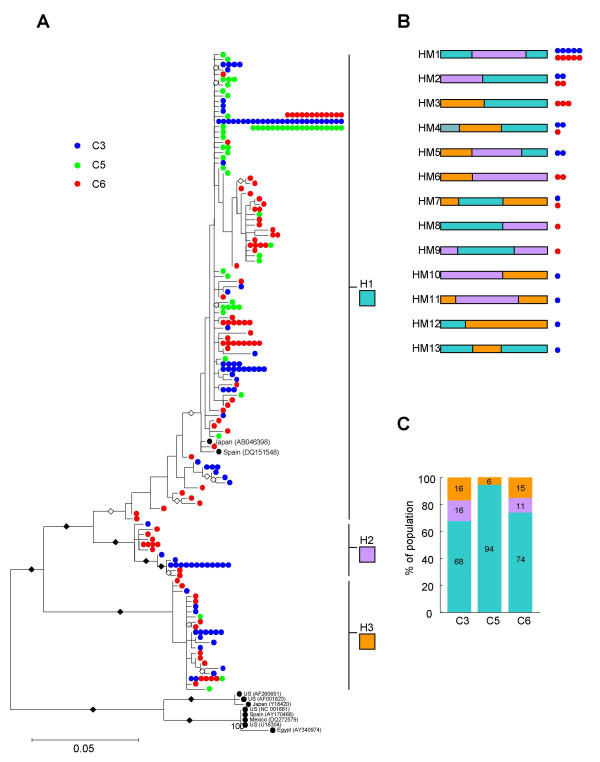
**Phylogenetic and recombination analysis of 284 HSP70h gene fragments sampled from three Brazilian orange trees**. (A) Largely recombination free maximum likelihood phylogeny of 252 HSP70h gene fragment sequences. Sequences sampled from trees C3, C5 and C6 are represented by blue, green and red dots respectively. Multiple dots on particular branches represent identical sequences. Whereas branches with more than 75% bootstrap support are labeled with filled black diamonds, those with between 50 and 74% support are labeled with unfilled black diamonds. Sequence lineages H1, H2, and H3 are indicated. (B) The mosaic structures of obviously recombinant sequences excluded from the phylogenetic analysis. The mosaics are colour coded according to the main sequence lineages from which they have probably been derived. Coloured dots beside the schematic representations of the recombinant sequences represent the numbers and origins of recombinant sequences sharing similar mosaics. (C) The relative population representation of different major HSP70h lineages identified in A.

Maximum likelihood trees constructed using the remaining 269 RdRp and 252 HSP70h sequences (Figures 1A and 2A) indicated that, with a few exceptions, the sequences obtained from all three orange trees respectively fell into four and three distinct (>75% bootstrap support) RdRp and HSP70h lineages. The lineages were arbitrarily named R1 through R4 for the RdRp sequences and H1 through H3 for the HSP70h sequences. The R1 lineage was further divided into R1a and R1b groups on the basis of there being >50% boostrap support for a small but distinct R1b lineage. Whereas the R1b, R4 and H2 lineages contained only sequences sampled from trees C3 and C6, the R2 lineage contained only sequences sampled from trees C5 and C6. There was no lineage containing only sequences sampled from the trees with CSD (C3 and C5).

We examined the inferred mosaic structures of the recombinant sequences identified previously and determined that most were clearly recombinants between the major lineages (Figs 1B and 2B). While we cannot rule out the possibility that some of these recombinants might be RT-PCR artifacts, the occurrence of nearly identical recombinant mosaics in different trees strongly suggests that at least three of the RdRp recombinants (RM1, RM3, and RM5) and four of the HSP70h recombinants (HM1, HM2, HM4, and HM7) are genuine. As none of these recombinants were found in both of the trees with CSD, they can also be excluded as causal agents of the disease.

Interestingly, all seven of these "shared" recombinants were detected in the tree without CSD, C6, and one of the trees with CSD (C3). Also, no HSP70h recombinants and only one class of RdRp recombinant (RM2) was found in C5, the other tree with CSD. One obvious reason for the lack of recombinants in tree C5 is that it was a much younger tree than the other two at the time of sampling. Analysis of the relative population representations of the two CTV gene lineages in the different trees (Figs 1C and 2C) also indicated that the CTV populations in the two older trees, C6 and C3 were substantially more similar to one another than either was to the CTV population in C5.

### There is no obvious association between CSD and CTV population structure

Genetic differences between CTV population structures in the three trees were quantified using Hudson, Boos and Kaplan's Kst* statistic [18]. Although CTV population structures were found to be significantly different between all trees (permutation test p-value < 0.001 for all tree and loci comparisons), the populations of both the RdRp and HSP70h sequences in trees C3 (with CSD) and C6 (without CSD) were substantially more similar to one another (Kst* = 0.037 for RdRp and 0.015 for HSP70h) than the populations in either tree were to populations sampled from tree C5 (for RdRp Kst* = 0.15 and 0.16 for comparisons of C5 with C3 and C6 respectively; for HSP70h Kst* = 0.042 and 0.051 for comparisons of C5 with C3 and C6 respectively). Unlike the association between CTV virulence and population structures observed by Sentandreu et al. [9], there therefore appears to be no simple association between population structures and CSD in these three Brazilian CTV trees.

## Conclusion

We have determined that the two sampled trees with CSD (C3 and C5) have more divergent population structures than one of these trees (C3) has compared to a tree without the disease (C6). Our analysis therefore suggests that, rather than a recombinant CTV variant or obvious differences between the population structures of mixed CTV infections, the most likely hypothesis relating CTV infections and CSD is that it may be caused by the occurrence and interaction of two or more specific CTV sequence variants within these mixed infections. If nothing else this study indicates that it may be extremely difficult to fulfill Koch's postulates for potential causes of CSD. Properly demonstrating that the existence of specific sequence variants within a mixed CTV infection are key to the development of CSD will require the construction of numerous infectious CTV full genome clones and a mechanism for artificially recreating complex mixed infections such as we've described here.

## Methods

### Virus sample collection

Three natural Brazilian CTV populations were surveyed. These were obtained from: A tree without CSD symptoms, C6 (12 years old), collected at Araraquara in São Paulo (SP) state and two trees with CSD symptoms, C3 (6 years old) and C5 (2 years old), collected at Colômbia, in SP state and at Comendador Gomes in Minas Gerais (MG) state, respectively. The three sampling locations were on an approximately 300 Km long straight line with C3 in the middle, approximately 200 Km from C6 and 100 Km from C5. Rootstock (Rangpur lime) phloem, specifically that showing yellowing symptom characteristic of CSD, was collected, initially stored on dry ice, and then frozen at -80°C until further processing.

### RNA extraction and reverse transcription-polymerase chain reaction (RT-PCR)

CTV genomic RNA was extracted using the RNeasy Plant Mini Kit (QUIAGEN, Valencia, CA) from triturated phloem parts with liquid nitrogen in a coffee grinder. Two pairs of specific RT-PCR primers were designed for amplification of a 542 bp RdRp fragment and a 564 bp HSP70h fragment using six complete CTV genome sequences [GenBank:U16304, GenBank:AF260651, GenBank:AB046398, GenBank:NC_001661, GenBank:AF001623, GenBank:Y18420]; RdRp-forward = GAA CCG GCT CGY GTT CGG CGT; RdRp-reverse = TTC CGC YAA CCC AGC GTT CGT CAT; HSP70h-forward = CTG GAG TTA TAT GTT CGG TAC C; HSP70h-reverse = ACG AGC TTC CAC CGA CTA CCA CTA. Total extracted RNA was denatured at 90°C for five minutes and reverse transcribed by incubation at 42°C for one hour in a reaction mixture with a total volume of 20 μl containing first strand buffer, 0.5 mM of the four deoxynucleoside triphosphates (dNTPs), 2.5 μM of reverse primer, 40 U of RNaseOUT (Invitrogen) and 200 U of reverse transcriptase SuperScript II (Invitrogen). An aliquot of 3 μl of this product was amplified in a 50 μl reaction mixture containing 1× buffer, 0.2 mM dNTPs, 2 mM MgCl_2_, 200 pM primers, and 1 U Platinum *Taq *DNA Polymerase High Fidelity (Invitrogen). The PCR conditions were: 94°C for 2 minutes, 35 cycles of each 94°C (1 minute), 55°C (1 minute), and 68°C (1 minute), and last extension of 72°C for 7 minutes.

### Cloning and sequencing of amplified fragments

RT-PCR amplified DNA fragments were separated by agarose gel, and DNA fraction was cut and purified using Perfectprep gel clearnup kit (Eppendorf). Adenosine residues were added to the 3' ends of purified DNA fragments using *Taq *Polymerase (Invitrogen) with dATP for 30 min at 70°C. Then, the DNA was cloned into pGEM-T (Promega) and transformed into *Escherichia coli *DH5α by electroporation [19]. For each of the two amplified gene regions plasmid DNA was purified from 300 transformed colonies using the Wizard Plus SV Minipreps DNA Purification System kit (Promega). Nucleotide sequences of these clones were determined in both directions using SP6 and T7 primers and an ABI Prism DNA sequencer 377 (Perkin-Elmer). Sequence quality control and individual sequence assemblies using reads from both strands were performed using the Staden package [20].

### Sequence analyses

RdRp and HSP70h fragments were aligned along with corresponding sequences from ten full length CTV genomes sampled from around the world ([GenBank:AF260651, GenBank:U16304, GenBank:NC_001661, GenBank:AF001623] from the United States, [GenBank:AY206452] from Israel, [GenBank:AB046398] from Japan, [GenBank:Y18420, GenBank:AY170468, GenBank:DQ151548] from Spain, [GenBank:DQ272579] from Mexico and [GenBank:AY340974] from Egypt) using the ClustalW method (with default settings) [21] implemented in Mega (version 3.1) [22]. Columns of the alignment containing indels or missing data for any of the sequences were excluded from analyses leaving indel-free alignments containing 499 and 411 columns of HSP70h and RdRp sequence respectively. Pair-wise Hamming distances between the sequences in each alignment were calculated using DNAMAN (Lynnon Corporation, Canada).

Recombination amongst the sequences in the two alignments was analyzed using the RDP [23], GENECONV [24], BOOTSCAN [25], MAXCHI [26], CHIMAERA [27], SISCAN [28] and 3SEQ [29] methods implemented in RDP3 [30]. Default settings were used throughout and only potential recombination events detected by two or more of the above methods coupled with phylogenetic evidence of recombination were considered significant. Also, the severity of Bonferroni correction during detection was minimized by only searching for recombinant signals in a single sequence within groups of sequences sharing >99.7% sequence identity.

Phylogenetic trees were constructed using the neighbor joining (JC distances, 1000 bootstrap replicates) and maximum likelihood methods (HKY model + Gamma with four substitution rates, transition/transversion ratios inferred from the data and 100 bootstrap replicates) implemented in MEGA and PHYML [31], respectively.

Genetic distances between populations sampled from different trees were estimated with DnaSP [32] using Hudson, Boos and Kaplan's Kst* statistic [18]. Significant differences between estimated Kst* values were determined with a permutation test (1000 replicates) also implemented in DnaSP.

## Competing interests

The author(s) declare that they have no competing interests.

## Authors' contributions

TN is the corresponding author and coordinated this project. DM contributed and TN, were involved in the statistical and recombination analysis of the data. Sample collection, gene cloning and sequence alignments were carried out by CG, WJ, CBN and GP. All authors have read and approved this manuscript.
